# The PHARMS (Patient Held Active Record of Medication Status) feasibility study: a research proposal

**DOI:** 10.1186/s13104-017-3118-3

**Published:** 2018-01-08

**Authors:** Elaine Walsh, Laura J. Sahm, Patricia M. Kearney, Henry Smithson, David M. Kerins, Chrys Ngwa, Ciara Fitzgerald, Stephen Mc Carthy, Eimear Connolly, Kieran Dalton, Derina Byrne, Megan Carey, Colin Bradley

**Affiliations:** 1 Department of General Practice, University College Cork, Western Gateway Building, Cork, Ireland; 2 School of Pharmacy, UCC, Mercy University Hospital, Cork, Ireland; 3 Department of Epidemiology and Public Health, UCC, Cork, Ireland; 4 Department of Pharmacology and Therapeutics, UCC, Mercy University Hospital, Cork, Ireland; 5 INSIGHT Centre for Data Analytics, UCC, Cork, Ireland; 6 Health Information Systems Research Centre, UCC, Cork, Ireland; 7 School of Medicine, UCC, Cork, Ireland; 8 School of Pharmacy, UCC, Cork, Ireland; 9 Mercy University Hospital, Cork, Ireland

**Keywords:** Medication error, Transitional care, Medication reconciliation, Information technology, Implementation science

## Abstract

Medication errors are a major source of preventable morbidity, mortality and cost and many occur at the times of hospital admission and discharge. Novel interventions (such as new methods of recording medication information and conducting medication reconciliation) are required to facilitate accurate transfer of medication information. With existing evidence supporting the use of information technology and the patient representing the one constant in the care process, an electronic patient held medication record may provide a solution. This study will assess the feasibility of introducing a patient held electronic medication record in primary and secondary care using the Consolidated Framework for Implementation Research (CFIR).This feasibility study is a mixed method study of community dwelling older adult patients admitted to an urban secondary care facility comprising a non-randomised intervention and qualitative interviews with key stakeholders. Outcomes of interest include clinical outcomes and process evaluation.This study will yield insights pertaining to feasibility, acceptability and participation for a more definitive evaluation of the intervention. The study also has the potential to contribute to knowledge of implementation of technology in a healthcare context and to the broader area of implementation science.

**Electronic supplementary material:**

The online version of this article (10.1186/s13104-017-3118-3) contains supplementary material, which is available to authorized users.

## Introduction

### Background

Medication errors are a major source of preventable morbidity, mortality and cost [1, 2]. Many errors occur during transitional care as patients move between different stages and settings of care [3]. Existing evidence suggests that medication errors frequently occur at the primary-secondary care interface, when patients move between the hospital and the community at time of admission to and discharge from hospital [4, 5]. Errors are particularly prevalent among elderly patients taking multiple medications [6, 7]. Key sources of potential error at this interface include legibility, documentation and communication between healthcare professionals [8–10].

Medication reconciliation is the formal process for identifying and correcting unintentional medication discrepancies during transitional care and is promoted as a method to improve patient safety internationally [11–13]. The goal of medication reconciliation is to provide the patient with an up to date and accurate list of medications that is available and is reviewed in all settings and stages of care [14]. Different strategies used for medication reconciliation include: a multidisciplinary approach [15]; pharmacist-led initiatives [16] and complex multifaceted interventions [17]. A recent systematic review identified a lack of consensus regarding the most effective method of medication reconciliation and a lack of reduction in healthcare utilisation among initiatives to date [18].

Novel interventions are required to optimise medication reconciliation and to address the issue of medication error at the primary–secondary care interface. There is evidence to suggest that information technology has a role in medication reconciliation [19–21], particularly medication reconciliation at the primary–secondary care interface [22]. General practitioners (GPs) are an accurate provider of patients’ medication information [23] and hence integration of medication information from primary care at time of hospital admission and discharge may facilitate medication reconciliation between primary and secondary care. In a consensus statement on medication reconciliation Greenwald et al. state that: “A personal health record that is integrated and easily transferable between sites of care is needed to facilitate successful medication reconciliation” [14]. The patient represents the one constant in care processes, including transitions in care, and hence an electronic patient held record of medication using medication information from primary care may be a viable option to assist in the process of medication reconciliation at the primary secondary care interface.

### Intervention development and evaluation

**Fig. 1:**
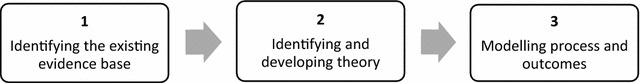
Steps of the development stage of a complex intervention outlined in the MRC methodological approach [26]

The process of developing and introducing a new technology in a healthcare context is complex. Though the benefits of technology in a healthcare context are well established, many interventions found to be effective in health services research fail to be successfully implemented and hence fail to improve patient care [24]. Barriers to implementation may occur at multiple levels: the patient level, the provider level, the organizational level or the policy level [25]. To address such issues, the UK Medical Research Council (MRC) recommends a structured methodological approach in developing a complex intervention in the healthcare setting. Systematic development is recommended based on best available evidence and appropriate theory [26]. The steps recommended in development are outlined in Fig. 1:

Following development, the MRC recommends testing of interventions in a phased approach beginning with a feasibility study and moving on to exploratory and finally definitive evaluation [26].

Existing evidence has identified both the need for novel interventions to assist with medication reconciliation and the potential of an electronic patient held medication record to provide a solution [14, 18, 19]. In accordance with such evidence and the MRC methodological framework a secure password protected electronic patient held medication record has been developed (Additional file 1). The device utilises the Universal Serial Bus (USB) port of any computer and provides a link to the patient’s general practice medication record. The device acts as a key providing access to a patient’s medication list as it appears in their general practice record with no information being stored on the device. Changes to a patient’s medication may be documented via this device in secondary care but the master list may only be altered by a patient’s GP in primary care.

Successful development and implementation of a novel intervention within the healthcare setting requires a detailed understanding of the context in which it is being introduced and potential barriers to implementation. The introduction of an electronic Patient Held Active Record of Medication status (PHARMS) at the interface of primary and secondary care involves multiple stakeholders (patients, healthcare professionals and information technology personnel), and two settings of care. To explore the issues surrounding development and implementation of this novel intervention prior to a definitive evaluation, in line with MRC guidance, the Consolidated Framework for Implementation Research (CFIR) will be used. The CFIR is a meta-theoretical framework. It combines key elements from published implementation theories and provides a structure to verify what works, where and why across multiple contexts. It consists of five domains. Each domain consists of factors and influences which impact the degree to which an intervention or practice is adopted [27]:

1. Intervention characteristics.
2. Outer setting.
3. Inner setting.
4. Characteristics of the individuals involved.
5. Process of Implementation.

### Study objective

This paper outlines the protocol for a feasibility study of introducing an electronic patient held medication record in primary and secondary care using the CFIR. The study aims to examine the performance of the device in addition to establishing acceptability of the initiative to key stakeholders and identifying the barriers and facilitators to the process of its implementation.

## Main text

### Methods

The CONSORT 2010 extension for pilot and feasibility trials has been used to inform study methodology [28].

Based on the MRC recommendation of testing an intervention in a phased approach, the study will be conducted in two phases as illustrated in Fig. 1.

Phase 1: introduction of the patient held electronic medication record at the interface of primary and secondary care at time of hospital discharge
Phase 2: introduction of the patient held electronic medication record at the interface of primary and secondary care at time of hospital admission.

**Fig. 2:**
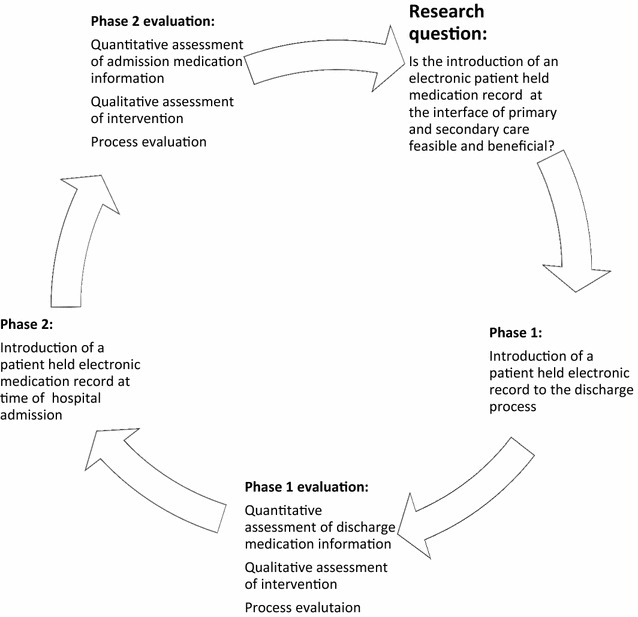
Phases of the feasibility study

The findings from Phase 1 will be used to inform the Phase 2 of the feasibility study as shown in Fig. 2.

### Study design

The feasibility study is a two phase non-randomised controlled intervention study with qualitative and quantitative evaluation components at each phase. Phase 1 will involve introduction of a patient held electronic medication record to the discharge process and Phase 2 will involve introduction of a patient held electronic medication record to the admission process. Realistic evaluation, informed by the approach used by Rycroft et al. [29], will be conducted by combining an experimental study design with exploratory research in order to identify issues pertaining to implementation.

### Setting

The study will be conducted in the five general medical and surgical wards of an urban 350 bed secondary care facility and four urban general practices. Following analysis of referral patterns over a 2-month period, the four practices were selected on the basis of having high rates of referral to the secondary care facility.

### Sample size

A sample size of 65 patients per arm has been calculated. Previous work in the same clinical setting has indicated a rate of 1.1 medication errors per prescription from a total of 1600 prescriptions written with in a similar timescale to that envisaged in the feasibility study [30]. A sample size of 65 from a population of 1600 prescriptions would be capable of providing estimates of the difference in medication error rates of 10% with a confidence of 90% [31].

### Participants

#### Patients

Community dwelling older adult patients (> 60 years) admitted to hospital who are taking three or more medications will be included in the study.

*Exclusion criteria* Patients who are resident in long-term care facilities, who are unable to provide written informed consent or who are in receipt of end of life care will be excluded from the study.

#### Staff

Hospital doctors and nurses, General Practitioners (GPs) and Information Technology (IT) staff will be involved in the study.

### Preparation and training

#### Healthcare professionals

Information regarding the study will be disseminated to all clinical staff of the participating secondary care facility via email in advance of commencing the study. The lead researcher (EW) will present at teaching sessions for medical, nursing and allied healthcare professional staff. Additionally, EW will provide two dedicated training sessions regarding use of the device to the non-consultant hospital doctors (NCHDs) of the secondary care facility and an education session to each of the four participating general practices.

#### IT

Software will be installed on one computer in each of the four participating general practices and on one computer on each of the five general hospital wards to enable the integration of the patient held medication record into the existing primary and secondary care IT systems.

The results of Phase 1 will be used to guide preparation for Phase 2. Further dissemination of information and education/training sessions will be provided as necessary for Phase 2.

### Phase 1: Introduction of the patient held active electronic medication record at time of hospital discharge

Eligible patients will be identified on all five general medical and surgical wards of the participating secondary care facility. Patients will be provided with an information leaflet and written informed consent will be obtained.

#### Intervention arm

Following hospital admission, eligible patients from the four participating GP practices will be assigned to receive an electronic patient held medication record. The device will be activated and the GP medication list will be linked from the electronic patient record in primary care. In order to maximise safety and confidentiality the chief investigator (EW) will be responsible for both transport of the device and linking the device to the patient’s record in general practice. General practice, community pharmacy, hospital admission and hospital discharge medication information will be collected on all patients and patient age, co-morbidities and functional status will be recorded. The patient is asked to retain the device during their inpatient stay. Intervention patients will be identified by a sticker on their medical notes, nursing notes and drug chart. A note will also be entered by the lead researcher (EW) into their medical and nursing notes. At time of discharge the patient’s hospital doctor will obtain the device from the patient. The doctor will use the device to access the patient’s pre admission medication list when generating the discharge prescription for the patient and to note/explain any alterations made during the inpatient stay. The discharge medication information and associated explanations of alterations will be communicated electronically to the patient’s file in general practice at time of generating the discharge prescription. This information will appear as a separate electronic document in the patient’s file in general practice. A discharge prescription will be printed for the patient and the device will be returned to the patient’s care at time of hospital discharge. Following discharge, the patient’s GP will be able to access the document in the patient’s file and adjust the master medication list accordingly.

#### Control arm

Following hospital admission eligible patients on all five general medical and surgical wards from non-intervention general practices will be identified. General practice, community pharmacy, hospital admission and hospital discharge medication information will be collected on all patients and age, co-morbidities and functional status will be recorded. At the time of discharge this patient group will receive usual care in the form of a handwritten discharge prescription.

### Phase 2: Introduction of the patient held electronic medication record at the interface of primary and secondary care at time of hospital admission

Eligible patients will be identified by their GP in the four participating GP practices at time of referral to the emergency department of the secondary care facility.

#### Intervention arm

GPs will issue patients with a device and activate the device at time of referral to the emergency department. The patient will be asked to retain the device and to present the device to the staff of the emergency department at time of arrival. The staff of the emergency department will use the device to access the patient’s pre-admission medication list. General practice, community pharmacy, hospital admission and hospital discharge medication information will be collected on all patients and age, co-morbidities and functional status will be recorded.

#### Control arm

Pre-admission and admission medication information data from Phase 1 control patients will be used.

### Outcomes of interest

Outcomes of interest reflect the CFIR domains and include both clinical outcomes and process evaluation. The outcomes are based on realistic evaluation firstly, to facilitate action research by using the findings of Phase 1 to inform Phase 2 and secondly, to develop explanatory theory to inform a future definitive evaluation. Table 1 outlines the CFIR domains and the relevant study measures.

**Table 1:** CFIR domains and relevant study measures

CFIR domains	How the domain aligns with the implementation of the electronic patient held medication record	Relevant study measures
Intervention characteristics	Relative advantage of device over usual practice Use of device (design and complexity)	Perceptions of hospital healthcare professional, GPs and patients regarding use of the device (qualitative interviews) Non-participant observation
Outer setting	Importance as perceived by wider secondary and primary care stakeholders Promotion of use of the device from clinical and administrative directors/leaders within the participating hospital and general practices	Perceptions of hospital healthcare professionals, GPs and patients regarding potential of the device (qualitative interviews) Occurrence of medication error (quantitative analysis of medication information) Non-participant observation
Inner setting	Readiness for change, quality of communication and teamwork within the participating hospital and general practices	Perceptions of hospital healthcare professionals, GPs and IT staff (qualitative interviews) Non-participant observation
Individual characteristics	Knowledge, beliefs and motivation of individuals involved in the study	Perceptions of hospital healthcare professionals, patients and GPs (qualitative interviews)
Implementation process	Establishing a plan for evaluation on a larger scale Methods to engage relevant individuals	Perceptions of hospital healthcare professionals, patients, GPs and IT staff (qualitative interviews) Non-participant observation

### Clinical outcomes

#### Phase 1

Prevalence of prescribing errors will be determined in both arms by evaluation of the discharge prescriptions.

Specifically, each prescription will be assessed regarding:

1. Patient demographics and legal requirements.

- Name and address.
- Date.
- Age or date of birth.
- Prescribers signature.
- Irish Medical council registration number for the prescribing physician.
- Controlled drug prescription requirements as specified by the Misuse of Drugs Act [32].

2. Therapeutics.

- Legibility.
- Accuracy of spelling.
- Presence of strength/dose/frequency.
- Quantity.
- Presence of repeat items and whether or not this is appropriate.
- Presence of drug–drug interactions as per Stockley’s Drug Interactions.

The prevalence of prescribing errors in the intervention and control groups will be compared.

#### Phase 2

Prevalence of discrepancies between the pre-admission medication information (GP and community pharmacy) and the admission medication information as prescribed on the patient’s hospital drug chart will be described.

In both phases data will be anonymised, coded and entered into an Excel spreadsheet on a password protected computer. Statistical analysis will be conducted using IBM SPSS version 24.

### Process evaluation

#### Qualitative interviews

Semi structured interviews will be conducted in Phase 1 and Phase 2 with:

1. Hospital healthcare professionals who used the device.
2. Patients who were issued with devices.
3. GPs of patients issued with devices.
4. IT professionals involved in primary and secondary care.

A census sample of hospital healthcare professionals, GPs and IT professionals will be sought. Sampling of patients will be purposive and will aim to ensure adequate representation of demographics such as age, gender and socioeconomic status. Descriptive statistics will be applied to demographic information. Interviews will be conducted until data saturation is reached [33].

Interviews will be recorded, transcribed and coded. Dual independent coding and thematic analysis will be conducted. Preliminary analysis will run concurrently with data collection and the topic guide will be amended as necessary. Data will be managed using N Vivo software on a password protected computer.

#### Non-participant observation

Direct observation of the implementation process will be conducted to identify barriers and facilitators [34]. Significant events such as medication interactions or omissions will be noted and medical staff of the participating care facilities informed. Observations will be recorded as field notes.

### Ethical issues

Written informed consent will be obtained from all participants. In view of the inclusion of potentially vulnerable older patients in the study, ability to provide informed consent will be assessed on a case by case basis, liaising with medical/nursing staff and family members where appropriate.

To limit any possible loss of confidential information, security has been a priority in device development and the device is protected to the highest level. Additionally, patient information accessed via the device has been limited to medication information.

## Discussion

Medication error during transitional care is an important patient safety issue and establishing effective medication reconciliation strategies is currently an international priority [11–13]. Benefit has been demonstrated with use of electronic systems of medication reconciliation during transitional care [19, 21, 22, 35]. Prior research has highlighted firstly the importance of integration of medication information between primary and secondary care [35] and secondly the need for multidisciplinary and patient involvement [35, 36]. To date there is no consensus regarding the most effective method [18] and the patient held electronic medication record represents a novel method of electronic medication reconciliation. It has the potential to harness the proven benefit of electronic medication reconciliation in addition to providing a novel method of integration of medication information between primary and secondary care. It has the additional potential to empower the patient within the medication reconciliation process. However, the development and implementation of a new technology in the healthcare setting at the interface of primary and secondary care presents multiple challenges. It is anticipated that the use of the CFIR in combination with clinical and realistic evaluation in this feasibility study will yield insights for a further more definitive evaluation of the electronic patient held medication record. The results of this study will be used to inform the design of a randomised controlled trial. Specifically, information will be obtained regarding feasibility, acceptability, participation rates and loss to follow up to inform a future trial. In addition, this study has the potential to contribute to knowledge of implementation of technology in a healthcare context and to the broader area of implementation science.

## Limitations

This feasibility study is a small scale study limited to a single secondary care site. Additionally, the intervention will not be introduced in community pharmacies in this study. Further work will be required to explore introduction of the device in multiple sites and the potential for use in the community pharmacy context.

## Additional file

**Additional file 1.** Patient held medication record. Additional detail on mechanism of patient held medication record.

## Abbreviations

PHARMS: Patient Held Active Record of Medication Status
GP: General Practitioner
CFIR: Consolidated Framework for Implementation Research
IT: information technology
MRC: Medical Research Council
NCHD: non consultant hospital doctor
