# Supplementary material for: The PHARMS (Patient Held Active Record of Medication Status) feasibility study: a research proposal
Source: BMC Res Notes. 2018 Jan 8;11:6. doi: 10.1186/s13104-017-3118-3 (PMC5759168; doi:10.1186/s13104-017-3118-3)

**Additional file 1**

**Patient held electronic medication record**

A secure password protected electronic patient held medication record which utilises the USB port of a computer has been developed through collaboration between the Department of General Practice University College Cork (UCC), GP software provider Si-Key Ltd, INSIGHT Centre for Data Analytics UCC, The Health Information Systems Research Centre UCC, the Tyndall Institute and the Technology Transfer Office, UCC.

Once activated, the device (Figure 3) provides a link to medication information in the patient’s record in general practice. Medication information from the patient’s general practice record may be viewed in secondary care by inserting the device into the USB port of a computer that has had relevant software installed.

In addition to providing a list of a patient’s pre-admission medication, the device has the facility to generate a discharge prescription. Changes to a patient’s medication may be documented in a dedicated note section when generating the discharge prescription. Both the prescription and notes regarding medication changes are transmitted electronically to the patient’s file in general practice and appear in the “Documents” section of the file. The medication list accessed by the device may only be altered by a patient’s GP in primary care.

Image of electronic patient held medication record:


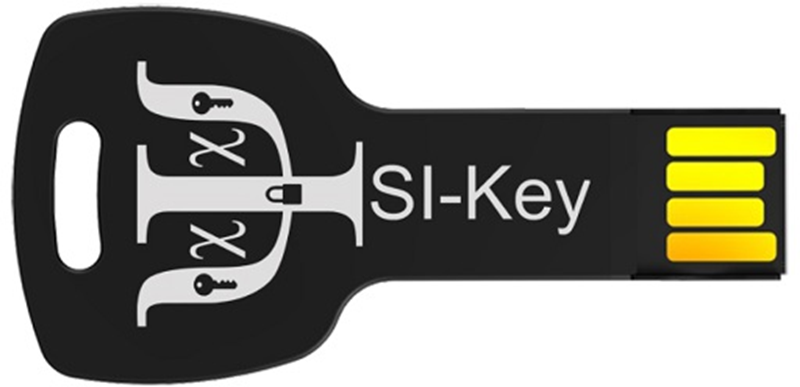

Supplement: Supplementary file 1 — Additional file 1. Patient held medication record. Additional detail on mechanism of patient held medication record. [file 13104_2017_3118_MOESM1_ESM.docx]
